# Scaling Lassa Virus Dynamics within Anthropogenic Ecosystems (SCAPES) Study Protocol: a mixed-methods observational cohort study of humans, rodents, and landscapes in Nigeria.

**DOI:** 10.12688/wellcomeopenres.25937.1

**Published:** 2026-02-16

**Authors:** Sagan Friant, David Simons, Christina Harden, Natalie Imirzian, Ottar Bjornstad, Rory Gibb, Kate Jones, Abigail Smith, Katharine Thompson, Aaron Lever, Fisayomi Aderibigbe, Wilfred Ayambem, Nzube Ifebueme, Helen Ignatius, James Koninga, Martin Meremikwu, Lina Moses, David Redding

**Affiliations:** 1 Department of Anthropology, The Pennsylvania State University,University Park, Pennsylvania, USA; 2 Huck Institutes for the Life Sciences, The Pennsylvania State University, University Park, Pennsylvania, USA; 3 Calabar Institute for Tropical Disease Research and Prevention, University of Calabar Teaching Hospital, Calabar, Cross River, Nigeria; 4 Center for Infectious Disease Dynamics, The Pennsylvania State University, University Park, Pennsylvania, USA; 5 Science Department, Natural History Museum, London, England, UK; 6 Department of Genetics, Evolution and Environment, University College London, London, England, UK; 7 Tulane University School of Public Health and Tropical Medicine, New Orleans, Louisiana, USA

**Keywords:** One Health, socio-ecological systems, zoonotic spillover, Mammarenavirus lassaense (LASV), West Africa

## Abstract

**Introduction:**

Zoonotic spillover driven by human activity remains a critical challenge. Research focusing on disease ecology or human–animal interactions provides detailed local insights, but these findings are often siloed and disconnected from global processes and policy. Risk maps are more commonly used to inform decisions, yet their connection to local-scale drivers of transmission remains poorly understood. This study bridges these gaps through a cross-scale study of Lassa fever, a rodent-borne hemorrhagic fever of public health significance in West Africa and a WHO priority pathogen. We use a fine-scale quantitative and participatory modelling approach that explicitly integrates into broad-scale risk models to identify the patterns and processes that drive spillover within human-driven ecosystems at the animal-human interface. Lassa fever epidemics are dominated by endemic and seasonal transmission of *Mammarenavirus lassaense* (LASV) from rodent reservoirs to humans within a rural context, positioning LASV as a uniquely tractable system in which to study zoonotic spillover.

**Methods and Analysis:**

We describe parallel longitudinal observational studies of humans, rodents, and landscapes that are explicitly designed to feed into a cross-scale quantitative modelling framework. By integrating data on landcover, rodent dynamics, human behavior, and LASV infection, we examine how anthropogenic activities shape LASV ecology and human exposure. These data will inform spatial models of the human-rodent-LASV interface to predict key drivers of exposure risk. Finally, data from these empirical studies and emergent patterns from the interface model(s) will be integrated into an existing broad-scale regression-based risk model. Together, our local-scale research and model predictions will clarify how zoonotic risk propagates from local to regional scales, providing evidence to inform disease management efforts for Lassa fever and zoonotic spillover more broadly.

## Plain Language summary

Lassa fever is a viral disease that spreads from animals to people and can cause serious illness in many parts of West Africa. The virus is mainly spread from rodents to humans, but it remains unclear why infection risk varies so much across places and communities. Everyday human activities, such as farming, food storage, housing, and land use change, shape where rodents live and how often people encounter them.

This study combines long-term data from humans, rodents, and landscapes in Nigeria to better understand how these interactions influence exposure to Lassa virus. We collect information on rodent populations, human behavior, land cover, and infection. These data are integrated into a central modeling framework that links fine-scale local interactions with broader patterns of disease risk. By connecting processes observed in communities to broad-scale risk maps, this cross-scale approach aims to improve understanding of where and why spillover occurs, and to support more effective, locally appropriate strategies to reduce Lassa fever transmission.

## Introduction

**Figure 1.**
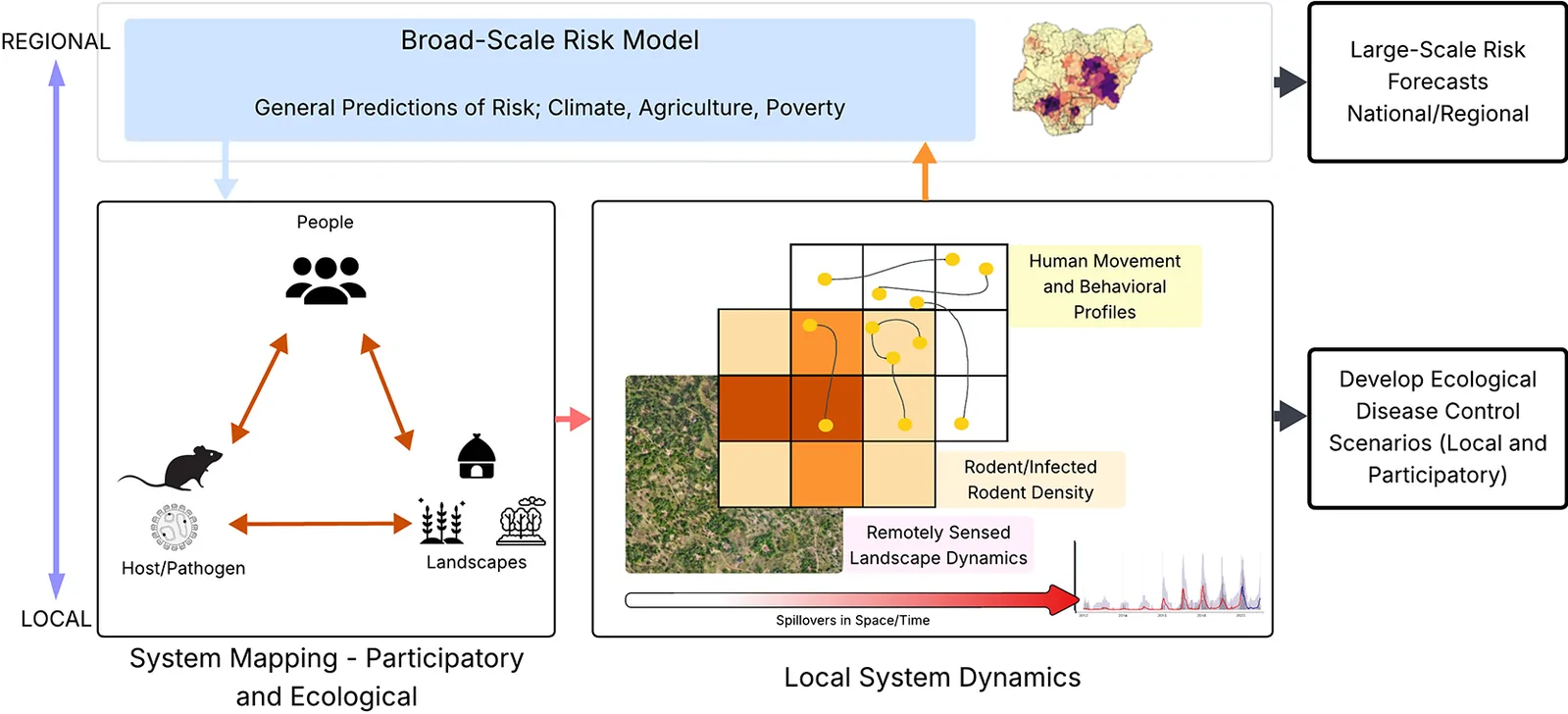
Cross-scale modelling framework. Local-scale data on human, rodent, and landscape factors are synthesized into socio-ecological motifs. These motifs inform interface models that identify the drivers of local transmission risk. The most robust drivers are then used to build a parsimonious, broad-scale predictive model. The final model is calibrated and validated using out-of-sample data from targeted field surveys in predicted high- and low-risk areas, ensuring the accuracy of the final risk maps.

Zoonotic spillover – the process by which a pathogen is transmitted from an animal to a human host – is a poorly understood phenomenon. Ecological and anthropological studies conducted at local scales are critical for understanding local-scale processes affecting spillover, but often lack the scope needed to explain global patterns of disease or to effectively inform policy decisions. Meanwhile, broad-scale (e.g. national or global-level) models forecasting spillover risk at policy relevant scales focus largely on correlates risk without an understanding of underlying socio and ecological spillover mechanisms. Discontinuities between broad and local-scale models have limited the explanatory potential of either approach. In many contexts, broad-scale models are limited by lack of detailed socio-ecological data (e.g., spatially consistent information on human and reservoir behavior), and local-scale studies are not designed to explicitly fill these gaps. A unified approach is needed to understand how broad-scale patterns of risk manifest in local transmission dynamics, and how local scale processes, in turn, scale up to shape broader patterns of disease ( Figure 1).

Lassa fever is a rodent-borne hemorrhagic fever of public health significance in West Africa and a WHO priority pathogen. ^1^ Like many emerging zoonoses, Lassa virus ( *Mammarenavirus lassaense*; LASV) spillover is characterized by multiple interacting species, spatial and temporal unpredictability, interacting biological and social factors, and difficulties in translating knowledge into management tools for public, agricultural, and ecosystem health. ^2^ The emergence and spread of LASV, as well as other zoonotic viruses, including hantaviruses, Nipah virus, Hendra virus, Ebola virus, and Marburg virus, is broadly associated with agricultural expansion and land use changes that alter the human-reservoir interface. ^3– 10^ Unlike other wildlife-origin zoonoses (e.g. Ebola, HIV, SARS-CoV-1 & 2) which occur in sporadic outbreaks with subsequent human-to-human transmission chains that are spatially and temporally removed from spillover events, ^11– 14^ LASV spills over frequently, with zoonotic transmission accounting for the majority of human infections ranging from asymptomatic infection to acute hemorrhagic fever. ^15– 17^ Lassa fever therefore provides a uniquely tractable system to elucidate the drivers of spillover within human-driven ecosystems.

*Mastomys natalensis* (the multimammate rat) is the primary reservoir for LASV, although the role of other infected, sympatric small-mammal species in viral transmission and maintenance remains poorly understood. ^18– 21^ *Mastomys natalensis* is abundant and widespread across sub-Saharan Africa, dominating agricultural landscapes, while LASV infection within these rodents is limited to their West African range and heterogeneous within it. ^2, 7, 22^ Human infections may occur via direct contact with infected rodent bodily fluids or indirectly through contact with contaminated food, surfaces, or particles. Risk factors that alter the likelihood, route, and dose of infection are putatively linked to housing, food processing and storage, pest management, and hunting and consumption of rodents ^23^; however, the exact transmission mechanism(s) and their underlying drivers remain poorly understood.

**Figure 2.**
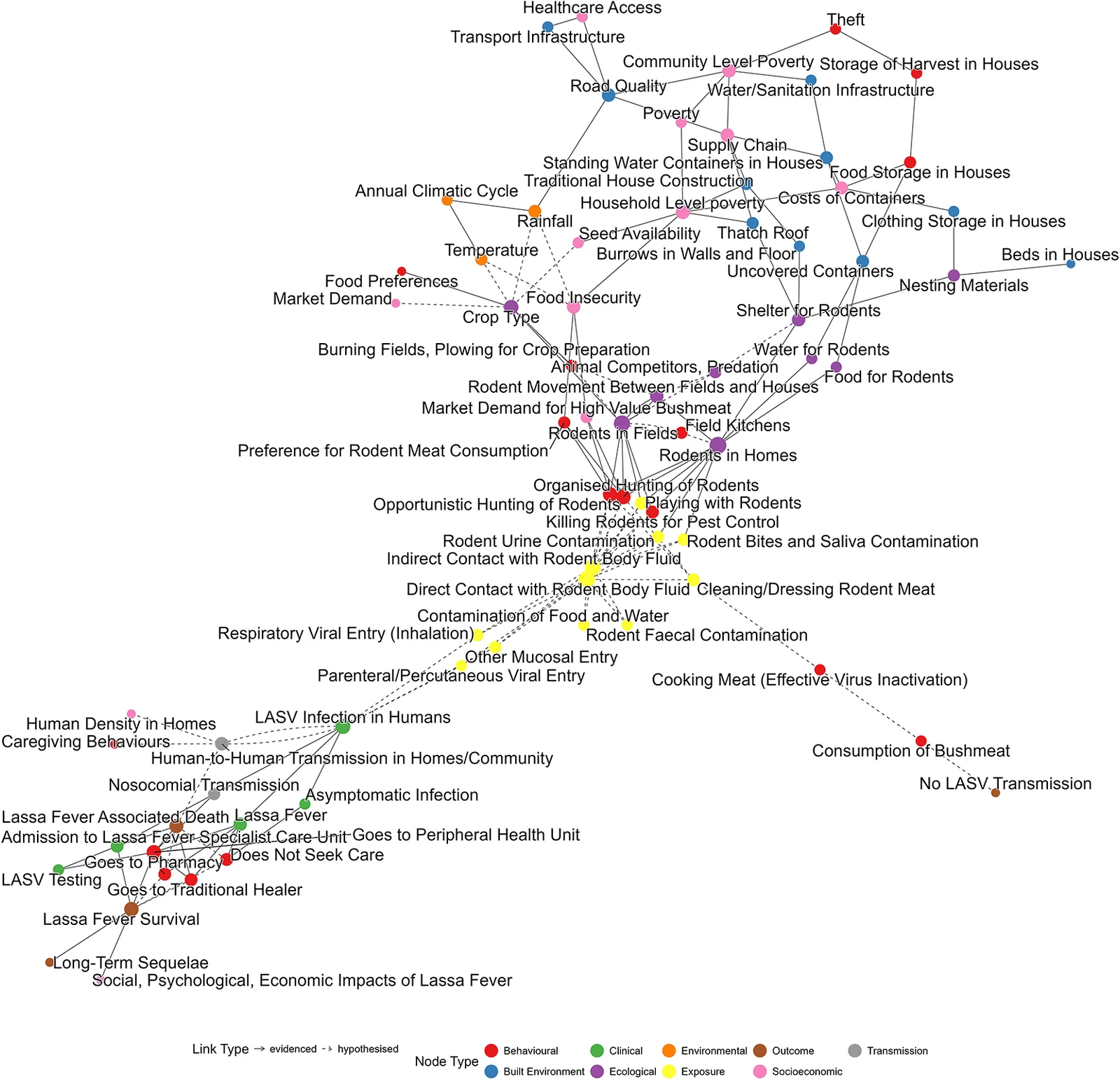
Conceptual model of the Lassa fever socio-ecological system. The network illustrates hypothesized causal pathways from upstream socio-ecological drivers to downstream clinical outcomes. Nodes are colored by conceptual link to Lassa fever and sized by their number of connections (degree centrality). Arrows indicate the direction of influence, while lines represent evidenced (solid) and hypothesized (dashed) links. For detailed annotations and references, see the interactive version of this model at the SCAPES project website.

To understand LASV risk, we must consider the complex socio-ecological systems in which spillover occurs ^24– 26^ ( Figure 2). Human activities influence LASV spillover through both top-down forces (e.g., rodent hunting) and bottom-up pressures (e.g., land use and agricultural practices) that shape rodent population dynamics and human exposure. In LASV endemic regions, the primary reservoir, *M. natalensis*, is simultaneously a food source, an agricultural and household pest, and a key driver of zoonotic disease. ^27– 29^ The social and ecological consequences of these interactions likely explain crucial epidemiological patterns, such as the seasonality of human outbreaks tied to crop cycles and rodent recruitment, and the clustering of infected rodent within specific households. ^30– 34^ However, these fine-scale socioecological dynamics, together with the drivers of local transmission, are missing from current approaches to assessing and predicting risk. Understanding transmission therefore requires an integrated perspective as human-rodent-environment interactions are deeply embedded in local livelihoods and food systems.

Current approaches using broad-scale risk maps have focused on drivers like climate, land use, and poverty to predict where and when outbreaks may occur. ^10^ While useful for high-level preparedness, these maps treat risk as an emergent property and are fundamentally limited because their inferential power is disconnected from the local-scale mechanisms that generate that risk. This scale mismatch, where policy is set broadly but interventions must be effective locally, hinders the development of targeted public health strategies. To truly understand and manage Lassa fever, and spillover infections more generally, we must bridge this gap by integrating knowledge of local processes across known axes of risk (e.g., rodent contact, climate, land-use and poverty) and throughout the range of LASV endemicity (i.e. from low-burden to high burden). Our project - **Sc**aling Lassa Virus Dynamics within **A**nthro **p**ogenic **E**co **s**ystems (SCAPES; website ^35^) - directly confronts this challenge by employing a framework ( Figure 1) to identify how emergent, broad-scale patterns of risk are generated from the collective fine-scale disease processes within human-driven environments.

### Aims and objectives

The goal of this study is to contribute to the long-term reduction in Lassa fever burden in West Africa by: advancing our understanding of the complex dynamics of LASV spillover within human-modified ecosystems in West Africa, isolating those specific social and ecological contexts where spillover is most likely to occur, and using this improved systems understanding to develop more effective tools for forecasting zoonotic spillover. The protocol is designed to achieve three interrelated aims, centered on a prospective, longitudinal analysis:

1) Elucidate the key pathways and dynamics linking landscapes, humans, and rodents, that give rise to ‘hotspots’ of Lassa virus transmission risk, using parallel field studies and integrated pattern-, process-, and participatory-based models to identify opportunities for disease control.
2) Predict risk of Lassa fever at larger spatial scales by integrating local-scale socio-ecological data into broad-scale risk models.
3) Communicate research findings to public health decision makers and local communities by co-developing intervention scenarios that foster evidence-based decision making and community engagement.

## Protocol

### Study design

**Figure 3.**
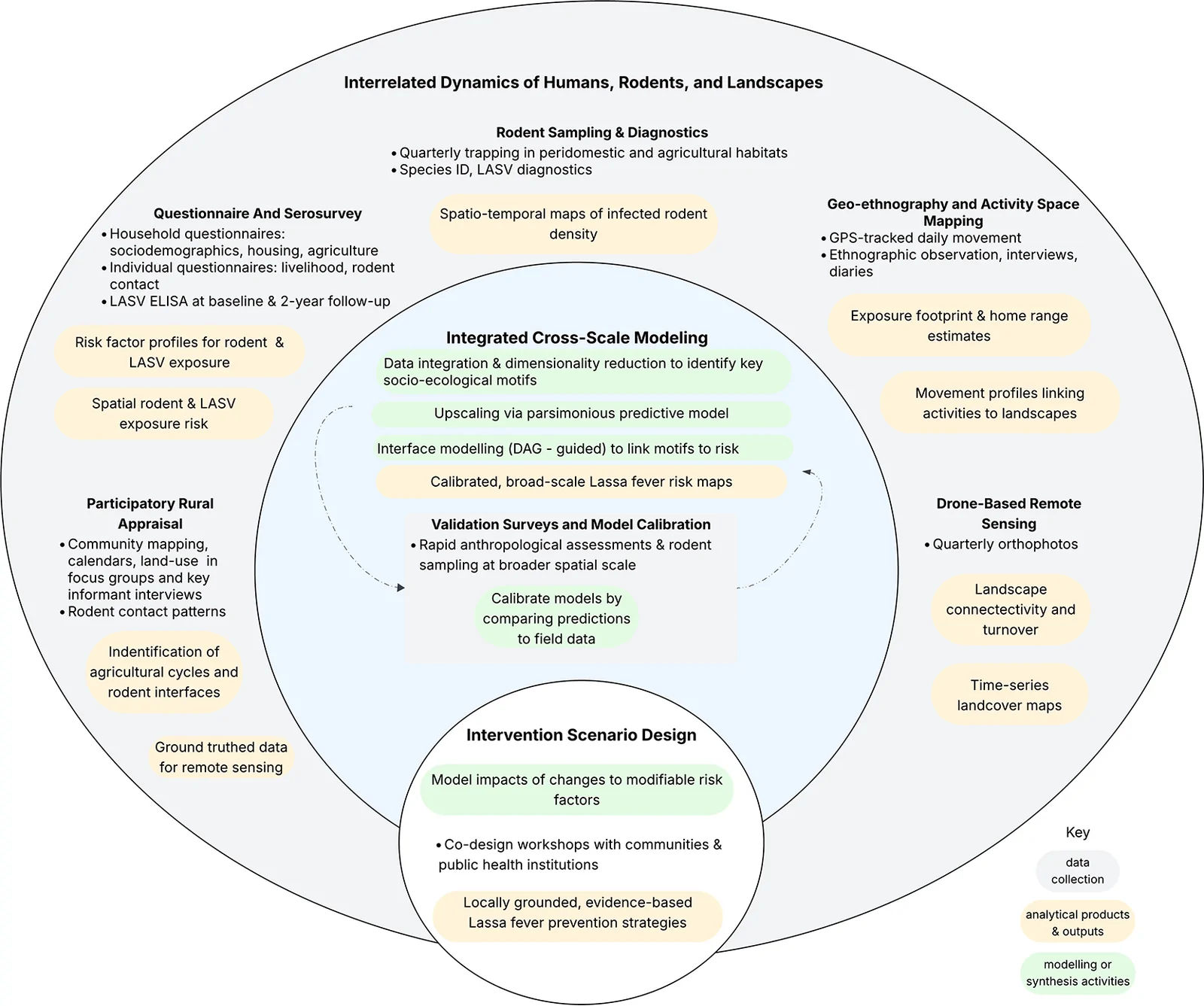
An integrated, cross-scale framework for the SCAPES project. The figure illustrates the project's workflow, structured as three nested stages moving from data collection and modelling to focused application. The outer grey area represents data collection conducted in aim one to characterize the *Interrelated Dynamics of Humans, Rodents and Landscapes*, where five interlinked components gather empirical data. The orange shading reflects the primary outputs of this aim - the generation of analytical data products. These products feed into the blue ring, the *Integrated Cross-Scale Modeling Framework*, where the green shading signifies the focus on modeling activities. The central white circle represents the final aim, the *Intervention Scenario Design.* Here we synthesize all findings into actionable strategies grounded in the communities and policy landscapes of the project.

The study will be conducted in selected sites within Nigeria, a country that reports the majority of Lassa fever cases but where disease risk is known to be markedly heterogeneous. ^10, 36^ Our field studies will be centered around three focal sites in Nigeria that encompass variation across broad-scale axes of risk and extend from Lassa fever hotspots to the edge of its predicted distribution. These local studies will incorporate epidemiological, anthropological, and ecological methodologies, tailored to capture the complex interplay between human activities, rodent reservoirs, and LASV transmission within human-driven landscapes ( Figure 3). Specifically, we will conduct a longitudinal study focused on anthropogenic landscapes, human movement and behavior, rodent ecology, and LASV surveillance. Our study is designed to collect parallel datasets needed to move beyond correlation and clarify the pathways that may drive virus transmission. These local-scale field studies will inform a set of interface models to examine the spatial and temporal distribution of LASV in rodent populations, identify when and where humans overlap with infected rodents, and characterize the behaviors that drive exposure in relation to dynamic landscape processes. Data from empirical studies and emergent patterns from the interface models will be integrated back into existing broad-scale regression-based risk models to predict risk at larger spatial scales. We will then conduct validation surveys at a broader spatial range to calibrate our predictive models.

### Study site and population

**Figure 4.**
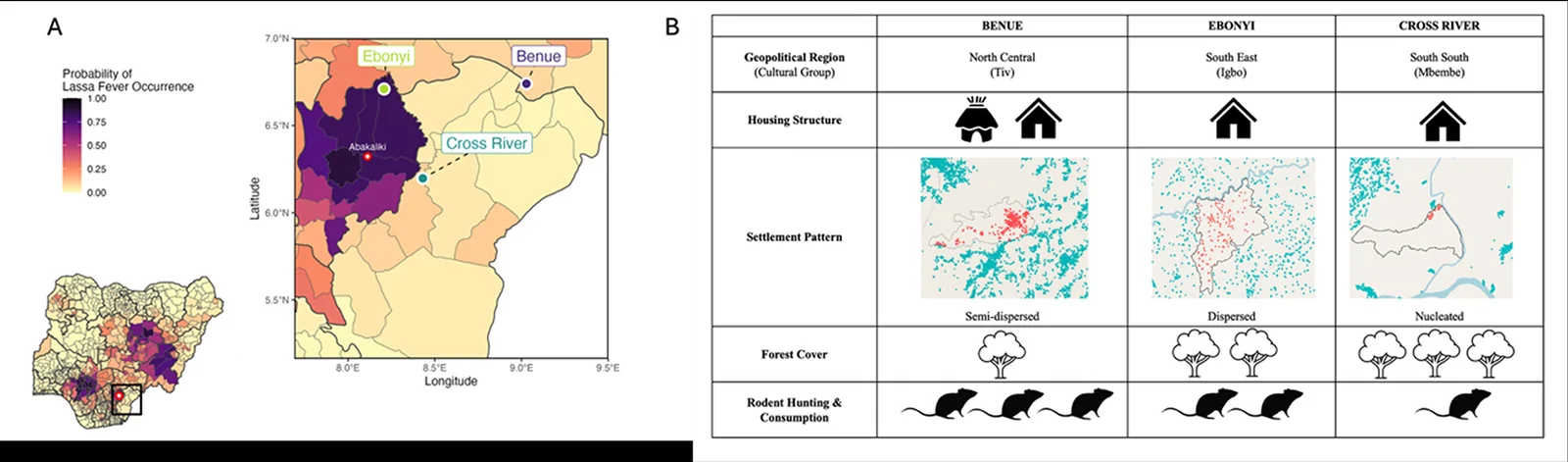
Study sites. The map shows the location of study villages within Nigeria and predicted probability of Lassa fever occurrence. Predicted probability values range from 0 (low; pale yellow) to 1 (high; dark purple) and are based on reported Lassa fever cases from 2018 to present (A). Socio-ecological conditions hypothesized to influence Lassa fever risk are expected to vary across sites. The three sites represent different geopolitical regions of Nigeria, dominant cultural groups, settlement patterns (e.g., spatially separated households surrounded by individual farmland vs. a single cluster of households with agricultural land spatially separated from villages), housing structures, land use practices, and cultural acceptance of small rodents as a food resource (B). Icons represent expected relative differences among sites rather than quantitative measures.

Our study areas span three states in Nigeria (Ebonyi, Benue, and Cross River) which vary in socioecological conditions expected to affect Lassa fever risk ( Figure 4). We identified specific study villages through a combination of cross-scale quantitative and qualitative assessments, integrating remotely sensed data with expert consultation and on-the-ground field visits to ensure local relevance and accuracy. Remotely sensed data were analyzed to select candidate field sites based on *a priori* quantitatively defined criteria: travel time (45 mins - 2 hours driving from our research base in Abakaliki, Ebonyi State), distance from major road (>1 km), village size (100-500 households), settlement patterns (nucleated vs. dispersed houses) and landcover (predominantly agricultural, with <60% forest areas). An initial list of potential villages was obtained from OpenStreetMap and supplemented using the Geographic Names Server maintained by the National Geospatial Intelligence Agency. ^37, 38^ Village size was estimated from the number of buildings using the Google OpenBuildings dataset. ^39^ Landcover characteristics, primarily proportion tree cover, cropland, grassland, and shrubland, were estimated through the ESA WorldCover dataset. ^40^ After identifying 426 villages within our region of interest, we narrowed down to 118 candidate sites based on the above quantitative criteria. We visually inspected sites on a satellite map to qualitatively assess whether they met our criteria. Shortlisted villages were assessed for feasibility, security, and reception to research through expert consultation at national, state, district, and community levels. Our quantitative criteria and expert consultation informed a hierarchy of villages based on accessibility or discordance with expected metrics. Study team members then conducted visits to candidate sites where they met with village heads and members from the community to assess fit (e.g., i.e., village size, land use types and major crops, and human-rodent interactions) as well as gather preliminary information on knowledge of rodents and Lassa fever. Ultimately, our focal sites were selected based on the receptiveness of the community to the research and research team to ensure that work could be conducted feasibly and safely.

### Ethics and dissemination

Human subjects research has been approved by the National Health Research Ethics Committee in Nigeria for Human Subjects Research (Protocol number NHREC/01/01/2007-17/07/2023), relevant State Ministries of Health, and the Pennsylvania State University Institutional Review Board (STUDY00019989). Animal research has been approved by the Nigeria National Veterinary Research Institute Animal Ethics Committee (AEC/03/168/24) and Tulane Institutional Animal Care and Use Committee (IACUC approval 2492). The research protocol adheres to principles of ethical community-based health research including fostering collaboration, enhancing transparency, supporting capacity building, and disseminating results to communities and public health authorities.

### Methods

AIM 1: Interrelated dynamics of humans, rodents, and landscapes

Cross sectional baseline study

Baseline data will include participatory rural appraisals, cross-sectional serological survey for LASV antibodies, and household and individual questionnaires. These data will be essential for classifying land cover and site boundaries, defining key variables, and constructing sampling frames, ensuring that the longitudinal study is feasible and that all models are empirically grounded and contextually informed. However, the cross-sectional nature of these data is insufficient to establish spatial temporality and identify the socio-ecological processes needed to inform effective interventions.

*Participatory rural appraisal*

We will use a collection of qualitative methods known as participatory rural appraisal to leverage local community knowledge of agricultural practices, land use, and human-rodent interfaces and ground our understanding of the socioecological system. ^41^ Within each focal village, we will conduct focus group discussions, key informant interviews, and transect walks to develop community maps and seasonal calendars. These exercises will elicit detailed community knowledge on the spatial and temporal patterns of agricultural and non-agricultural land use, rodent ecology, and activities that mediate human-rodent contact. Community derived land cover classifications and timing of agricultural cycles (e.g., clearing, planting, harvesting) will be used to define micro-zones of land use as well as ground truth and annotate features on our remotely sense maps. Local knowledge of human-rodent interfaces will inform the selection of key variables and the relationships between them, guiding both the causal structure and variable construction for our local-scale interface models.

*Questionnaire and serosurvey*

To identify the human behavioral and ecological factors that drive LASV transmission, we will conduct a mixed-methods, cross-sectional study across nine villages in our three study areas. A multi-module structured questionnaire will be administered to collect geocoded data on hypothesized risk factors. The instrument, informed by a systematic review of zoonotic disease literature and our participatory rural appraisal, will cover household sociodemographics, livelihood and agricultural practices, housing structure and location within the landscape, food storage methods, and the nature and frequency of human-rodent interactions. Specific modules quantified food security, rodent-driven food loss, and documented rodent off-take for consumption or pest control. Using systematic random sampling, we will enroll approximately 25% of households per village, targeting a total sample of 540 households for our quantitative questionnaire. Based on published and unpublished data, this sample size provides over 80% power (α = 0.05) to detect statistically significant differences in seroprevalence between villages classified as high-risk (~40% seroprevalence), medium-risk (~20%), and low-risk (<5%). ^42^ Within each household, we will seek to enroll an adult male, an adult female, and a child (≥12 years) for individual questionnaires and serology, plus an additional child (<12 years) for serology only, yielding an estimated 1,620 completed individual questionnaires.

As most LASV infections are subclinical, serological data are essential for accurately assessing cumulative exposure. Consenting participants will provide a finger-prick blood sample collected as a dried blood spot on filter paper, yielding an estimated 2,160 samples for analysis. These samples were tested for LASV-specific IgG antibodies using a commercially available ELISA (Panadea ^®^ LASV IgG), providing a direct measure of past infection. ^43^ These data also serve as a critical baseline for our ethnography cohort and for identifying risk factors for incident infections occurring during our longitudinal study.

Longitudinal parallel study

While our baseline data describe human–rodent interfaces, they cannot capture the spatial and temporal complexity, documented through community knowledge, and required to understand causal drivers. The longitudinal protocol therefore pursues three aims: defining these drivers, scaling them across landscapes, and translating findings into actionable interventions.

*Drone-based remote sensing*

To characterize the fine-scale landscape dynamics that structure human-rodent interactions, we will conduct quarterly drone-based remote sensing in each of the three focal villages. We will use DJI Mini 4 Pro (DJI, Shenzhen, China) to capture images at a 4K resolution (3840 x 2160 pixels). The drone will complete a full aerial survey in each village by flying back and forth in a lawn mower pattern. The drone will be flown at a height of 100m above ground and with ~25m between flight lanes allowing for over 70% side overlap between images. Images will be taken every 2 seconds to allow 75-80% front overlap, which provides sufficient overlap for reconstructing into composite orthomosaics. Collected images will be processed into high-resolution orthomosaics for each village using OpenDroneMap, ^44^ which uses feature matching to stitch images together. The spatial extent of these mapping campaigns will be guided by village boundaries established during our participatory rural appraisals.

From these orthomosaics, we will generate detailed land cover masks using a semantic segmentation approach. A subset of cropped areas will be taken from the orthomosaics from each village, chosen to contain sufficient representation of the land cover types of interest. We will focus on three main classes: forest areas, crop areas, and built-up areas. The cropped subset areas will be used to create pixel-level masks for training data using a semi-automated procedure with the Segment-Anything extension within CVAT. ^45^ Using these annotated masks, we will train a deep learning model to segment the land cover areas of interest. We will evaluate model performance by computing overall pixel accuracy, Intersection over Union (IoU) and F1 score on a held-out test set. After achieving sufficient performance, the model will be used to create land cover maps across the entire village. These high-resolution land cover maps provide a foundational geospatial framework for integrating all project data streams. They will be used to characterize “exposure surfaces”—spatial datasets capturing building location/material, land use, crop stage, and the structural complexity of agroecosystems. ^46^ We will then calculate spatial measures of connectivity between fields, houses and other key landscape features, as well as spatially explicit summaries of landscape complexity, turnover and evenness within focal buffers of 1m, 5m and 10m to serve as covariates in our interface models.

*Geo-ethnography*

To characterize the spatiotemporal dynamics of human behavior that mediate LASV exposure risk, we will conduct a longitudinal geo-ethnography. ^47^ This mixed-methods approach integrates high-resolution human movement data with mixed-methods ethnographic data collection to understand not just where people go, but also what they do there that might lead to rodent contact and ultimately LASV exposure. A spatially stratified random subsample of approximately 30 households (~90 individuals representing triads of men, women, and children from each household) across our three focal villages will be enrolled in this component and followed quarterly. Participants will carry a GPS tracking device (i-GotU GT-600B, 30s polling frequency) for 7 days each quarter to map high-resolution movement patterns. These tracks will be contextualized using data from daily activity logs and 7-day recall questionnaires recording major activities (e.g. planting, tending to and harvesting various crops, capturing wild animals etc.) and human-rodent interactions (e.g., sightings, indirect contact, direct contact) within visited locations.

For the qualitative components, we will conduct participant observations and key informant interviews with a subset of participants, reflecting diverse different sociodemographic backgrounds, livelihoods, and specialist knowledge. During participant observations, we will shadow purposively selected participants with different land use practices during their daily activities to document the timing, setting, and behavioral context of potential rodent contact events. These methods will deepen and contextualize our quantitative findings by capturing diverse experiences and perspectives at the human-rodent interface.

By documenting and geocoding the daily routines that shape the human–rodent interface, our analytical framework unifies individual-level behavioral data with landscape dynamics, fusing quantitative and qualitative data streams. We will first process the GPS data to quantify individual space use by calculating activity space measures (e.g., recursion metrics to identify frequently visited locations) and estimating individual home ranges and intensity distributions using autocorrelated kernel density estimation. ^48– 50^ These spatial footprints will be linked to our high-resolution land cover maps and systematically annotated with daily activity logs, weekly recall questionnaires, and ethnographic field notes to derive individual “exposure footprints” from GPS data for each quarter. This integrated dataset will allow us to produce heat maps that quantify the time spent in different land cover types (e.g., farms, forests, villages) and characterize: (1) the associated activities (e.g., crop processing and storage, hunting, cleaning) and (2) intensity and nature of reported rodent encounters within each context. These descriptive analyses will characterize seasonal variation in space use and associated activities. We will then aggregate individual movement tracks to examine the social (e.g. gender, occupation) and environmental (e.g. seasonality, landscape dynamics) drivers of community-level space use using integrated step selection functions. ^51^ Finally, using feature-based clustering, we will derive a minimum set of socio-ecological movement profiles that capture key variations in space use, behavior, and social context across the study population. These profiles will serve as a critical intermediate product, linking landscape data, questionnaire responses, and LASV exposure within our final risk models.

To link these detailed behavioral and movement profiles to our primary health outcome, we will capture longitudinal infection dynamics within this geo-ethnography cohort. Replicate serological samples will be collected from all participating individuals at the conclusion of our longitudinal study. This will allow us to measure seroconversion and seroreversion rates, providing a direct measure of incident infection. These incidence data will serve as the key outcome variable in our statistical models informed by causal reasoning, enabling us to test which specific socio-ecological profiles and movement patterns are the strongest predictors of new infections.

*Rodent sampling and diagnostics*

To characterize the reservoir component of the transmission system, we will conduct a parallel longitudinal rodent study to estimate the abundance, distribution, and LASV infection in the small mammal community. Our design is spatially stratified by land cover types defined by our remote sensing and participatory mapping data, focusing on two key strata: 1) the agricultural and forest matrix, and 2) the peridomestic environment. In the agricultural/forest matrix, we will establish six 10x10 trapping grids, each with 50 traps. ^52, 53^ In the peridomestic environment, we will place approximately 400 traps within and around the households enrolled in our geo-ethnography cohort. We will use a mix of smaller Sherman and larger Tomahawk live traps to capture a wide diversity of rodent species. Trapping will be conducted for four consecutive nights each session, yielding a total trapping effort of approximately 86,400 trap-nights over the course of the study.

All captured animals will be processed in a field laboratory following appropriate biosafety procedures. ^54^ For each individual, we will record demographic and morphological data and collect tissue samples (ear, blood, organs) for species identification (cytochrome-b PCR ^55^), age estimation (morphometrics and DNA methylation ^56^), and LASV diagnostics. Viral RNA will be detected using RT-PCR (RealStar ^®^ Lassa Virus RT-PCR Kit 2.0), and previous exposure will be assessed by testing for LASV-specific antibodies using the same ELISA protocol as for human samples. ^43^

To analyze these data, we will implement a spatial, multi-species N-mixture model within a Bayesian framework, using the spAbundance R package. ^57^ This package is specifically designed for hierarchical abundance modeling from repeated count data. This joint species distribution model (JSDM) framework will allow us to co-model the true, latent abundance and distribution of *M. natalensis* and other sympatric species, while simultaneously estimating species-specific detection probability. LASV infection status (PCR or antibody-positive) will be modeled as a secondary, conditional process. The model will estimate species-specific infection prevalence as a function of intrinsic factors (e.g., species, age class) and environmental covariates, conditional on the estimated abundance of each species at a given site.

Key covariates in both the abundance and infection models will include, landscape features derived from our drone-based data, seasonal drivers (e.g., rainfall, temperature, crop maturation) and interspecific factors (e.g., the abundance of competing species). The primary output of this analysis will be predictive, spatially explicit maps of infected and non-infected rodent abundance (and density) for each sampling period, including uncertainty estimates, which are essential inputs for our interface models.

AIM 2: Integrated cross-scale modelling framework

The central analytical goal of this project is to integrate our landscape, human, and rodent data to develop a cross-scale model of LASV transmission risk. This framework is designed to move beyond simple correlative risk models to identify and incorporate the key socio-ecological pathways underlying transmission into validated, predictive risk maps. The process involves three main stages: 1) data integration and dimensionality reduction; 2) interface modelling and scaling; and 3) out-of-sample model validation and calibration.

*Data integration and dimensionality reduction*

To enable cross-disciplinary analyses, we will first integrate all quantitative data (e.g., human behaviour, landscape, and rodent metrics) and coded qualitative data into a unified analytic dataset with standardized variable formats, coding schemes, and measurement scales. To identify the most salient patterns driving variance in our dataset, we will then apply unsupervised machine learning techniques, such as non-negative matrix factorization. This approach will reduce the dataset’s dimensionality by extracting key latent variables or “motifs” (i.e., distinct socio-ecological profiles of behaviour or specific landscape configurations) that account for most of the variance. ^58^ These derived motifs will serve as powerful, composite predictor variables in subsequent interface models.

*Interface modelling and scaling*

Our core quantitative analysis will employ Bayesian mixed-effects models to integrate the various data streams. To explicitly model the human-rodent interface, we will construct joint models that link spatiotemporal landscape dynamics with layers of predicted infectious and non-infectious rodent density, and human movement profiles. These models will allow us to estimate the effects of individual-level variables (e.g., age, sex, livelihood), household-level factors (e.g., housing materials, wealth), and dynamic behavioural variables (e.g., time spent in specific agricultural areas) on an individual’s risk of LASV exposure in space and time. A key feature of our Bayesian approach will be the incorporation of informative priors derived from our qualitative research, ensuring our statistical models are grounded in the socio-cultural realities observed in the field. ^59^

All quantitative analyses will be guided by a formal causal inference framework. For each primary hypothesis, we will first develop a Directed Acyclic Graph (DAG) based on an *a priori* conceptualisation of the socio-ecological system informed by the participatory rural appraisals and reviews of the published literature ( Figure 2). The purpose of each DAG is to make our hypothesized causal structures explicit, allowing us to distinguish between different types of covariates. ^60^ This process is critical for developing statistical models that minimize bias in estimating associations between exposures and outcome by identifying: confounders (variables that are a common cause of both the exposure and outcome that must be adjusted for), colliders (variables that are a common effect of the exposure and another variable that must not be adjusted for), and mediators (variables that lie on the causal pathway). Following these principles, our final regression models will adjust for the minimal sufficient set of confounding variables identified in each DAG, improving the validity and interpretability of our estimates.

The predictive performance and robustness of our models will be initially assessed through a multi-pronged validation strategy. ^61^ Cross-validation techniques will assess model stability by applying our model to different spatial patches and time periods within our primary study sites. We will then compare model predictions with observed Lassa fever case data. Specifically, we will test the model's capacity to reproduce the characteristic seasonal pulse of human infections and validate predictions against contemporaneous, district-level case surveillance data from the Nigeria Centre for Disease Control and observed seroconversion patterns in our cohort. ^62^ By testing these models at varying levels of data aggregation - from detailed local scales to broader regional scales - we can assess the scalability of our findings and the impact of spatial resolution on our understanding of LASV dynamics.

Following validation, we will develop a parsimonious predictive model of Lassa fever risk that can be applied across larger spatial and temporal scales. This model will incorporate the most stable and robust drivers identified through our earlier interface models, enabling the generation of broad-scale risk maps that are both empirically grounded and mechanistically informed. These maps will serve as tools for upscaling our findings, guiding further testing and refinement, and ultimately targeting interventions.

*Validation surveys and model calibration*

To validate and calibrate our predictive models with independent, out-of-sample data, we will conduct targeted “light touch” field surveys across a broader spatial scale, spanning a gradient of high to low predicted Lassa fever risk to observe whether our model predictions match local conditions. Using a risk stratified design, we will select 12 new villages based on model predictions, spanning predicted high-risk “hotspots” and low-risk “coldspots.” In each village, we will carry out rapid, concurrent data collection, including rodent trapping to assess *M. natalensis* presence and LASV prevalence as well as rapid anthropological assessments to characterize local human-rodent interfaces. This phase directly addresses a fundamental bias in LASV eco-epidemiology - the historical concentration of surveillance in areas with known human disease.

We will apply a standardized trapping effort in each village. This effort target will be quantitatively determined using rarefaction analyses on our longitudinal rodent data to identify the minimum trap-nights required to reliably characterize rodent community composition and detect LASV. The sampling design will mirror our longitudinal study, stratified between peridomestic and agricultural settings. As we will not have established participatory rural appraisal data for these new sites, specific trapping locations (households and fields) will be selected *a priori* using our trained predictive models, matched to remotely sensed landscape features to ensure key environmental variability is captured. All diagnostic testing will follow the RT-PCR and ELISA protocols described in Aim 1.

To characterize interfaces in each village, we will implement rapid anthropological assessments ^63^ that combine mixed-gender focus group discussions with targeted key-informant activities to capture the fine-scale social, spatial, and temporal dynamics of human–rodent contact. With support from community leaders, we will convene one light-touch focus group discussion per village, adapted from our prior participatory rural appraisal approach to elicit knowledge on human-rodent interactions, how interactions vary across landscapes and seasons, and how community knowledge aligns with or challenges outputs from our longitudinal study and interface models. Participants will review synthesized visualizations of modelled interfaces to identify areas of concordance or discrepancy with local conditions. From each focus group discussion, we will identify individuals demonstrating detailed or place-specific knowledge to join brief transect walks, spot mapping exercises, or key-informant interviews. Transects and spot maps will prioritize a diversity of places including, crop fields, storage areas, and ecological transition zones that are identified as important interfaces during focus group discussions. During these walks, participants will annotate spaces and discuss where and when rodent encounters are likely based on lived experience. Key-informant interviews we will deepen and clarify these insights, exploring sociocultural drivers of rodent contact, including gendered livelihood activities, land-use and tenure, settlement patterns and history, agricultural practices and commercialization, and governance or resource-access norms that may shape exposure risk. This streamlined, iterative design mirrors the logic of participatory rural appraisals while reducing field burden, enabling rapid triangulation between ecological data, modeled interfaces, and local experience to refine spatially and temporally explicit hypotheses about Lassa virus transmission.

The primary analysis will compare observed field data to the model's *a priori* predictions for each site. This approach allows us to rigorously test model performance and assess which drivers best explain discrepancies. For example, if LASV in rodents is confined to predicted hotspots, it would suggest that reservoir ecology is the primary driver of risk. Conversely, widespread LASV presence regardless of predicted risk would point to variation in human-rodent interfaces as the key determinant. Findings from this validation phase will guide the development of a final, parsimonious predictive model with improved accuracy and ability to generate reliable, spatially explicit risk maps.

AIM 3: Intervention scenario design and testing

AIM 3 applies a participatory scenario modelling framework to integrate local priorities, empirical model outputs, and co-designed intervention strategies to reduce LASV spillover. Participatory scenario modelling provides a structured approach for combining community-derived decision logics with model-based exploration of alternative system states, ensuring that planned interventions are both epidemiologically credible and locally feasible. ^64– 66^ All exercises will follow a consistent structure: small-group discussions (6–10 participants, stratified by age and gender) and in-depth interviews, audio recording, verbatim transcription, and iterative coding in NVivo. We will work in the same communities and aim to recruit the same groups from our preliminary participatory rural appraisal work — leveraging their existing familiarity with our team, data sources, and study goals.

*Criteria elicitation*

We will begin with a preparatory risk prioritization component that evaluates how participants prioritize competing rodent-related risks, such as disease avoidance, against rodent associated food and economic security, and how these priorities shape behavior relevant to LASV exposure. Drawing from participatory risk mapping, risk ranking, and multi-criteria analysis, we will use: 1) pile sorting and ranking exercises to compare perceived threats such as illness, loss of food and belongings, crop damage, and household contamination; 2) weighting tasks to assess perceived severity, frequency, and controllability; and 3) cognitive probes to elicit underlying rationales. Qualitative responses will be coded *in vivo* and inductively clustered into higher-order themes capturing emic tradeoff logics between health protection and livelihood preservation. These themes will be mapped onto behavioral constructs (e.g., attitudes, subjective norms, perceived behavioral control, and behavioral intentions) to clarify how competing priorities shape rodent contact or avoidance. Outputs will serve as the value- and criteria-based foundation for all scenario work, ensuring that scenarios reflect meaningful local priorities rather than externally imposed assumptions.

*Scenario generation*

Using insights from Aims 1–2 and risk prioritization logic, we will generate counterfactual scenarios—structured “what-if” comparisons that explore how modifying specific local conditions might have altered observed patterns of human–rodent contact or LASV spillover. Examples include changes in food-storage practices, rodent hunting methods, or agricultural practices that modify rodent habitats. Counterfactual analysis is widely used in infectious-disease modelling to isolate causal pathways and evaluate which changes produce meaningful reductions in risk. ^67^ In participatory scenario modelling, this component corresponds to exploratory scenario generation, in which empirical models simulate alternative system states that communities can then deliberate upon using vignette-based elicitation and narrative scenario prompts. Grounding counterfactuals in emic decision logics ensures they are both relevant, interpretable and aligned with lived experiences.

*Scenario deliberation and co-design*

Counterfactual scenario outputs will be used as inputs for community deliberation and collaborative intervention design. Discussions will include: 1) model walkthroughs that review model outputs and interpret emergent patterns in rodent populations, human-rodent interfaces, and land-use dynamics, and 2) ripple effect mapping to assess feasibility, acceptability and potential co-benefits of proposed control scenarios. Inductive and emergent themes will be linked with categories from Aims 1-2 (rodent abundance, human behavior, land use) to identify high-leverage, modifiable components to the Lassa system. Interventions with clear co-benefits are expected to be the most acceptable and sustainable. To further evaluate their feasibility, the interventions with the clearest community buy-in will be communicated during stakeholder workshops with local authorities, such as NCDC, state-authorities, and community leaders. These workshops will use pre-mortem analysis – an implementation science tool that anticipates logistical, economic, and governance obstacles prior to field deployment. ^68^ Findings will be disseminated through national-level reports and workshops as well as community meetings, to ensure transparency and enable iterative feedback.

## Discussion

The transmission of animal diseases to humans has significant consequences for public health and economies globally. When evaluating disease risks, policy decisions often rely on data visualization techniques, such as risk maps. However, there is a limited understanding of how broad-scale risk maps relate to local-scale processes that affect disease transmission from animals to humans. For example, risk maps are often missing vital information on how broad associations between disease risk and factors such as climate, land-use, and poverty are driven by interactions between humans, animals, and the environment that affect probability of human infection. On the other hand, localized studies revealing fine-scale disease processes are often poorly situated to inform projections of risk at broader spatial scales. This project bridges this gap through a study of Lassa fever, a rodent-borne hemorrhagic fever of public health significance in West Africa and a global health priority.

Lassa fever outbreaks are a yearly occurrence in Nigeria. There is currently no vaccine, and prevention of Lassa fever is dependent on early diagnosis and managing the human-reservoir interface. However, effective management of this interface is hampered by a limited understanding of the nature and extent of human-rodent interactions across West Africa. This knowledge gap is compounded by a historical sampling bias, as LASV surveillance has largely been concentrated in recognized ‘hotspots’. Consequently, our ability to forecast risk for LASV and other endemic rodent-borne infectious diseases outside of these ‘known knowns’, and to develop socially and ecologically based interventions to prevent spillover remains limited. A major strength of this study is that our sampling strategy is explicitly designed to address this limitation, providing a more grounded understanding of spatial variation in risk.

Field studies within SCAPES sample across known broad-scale drivers of Lassa fever risk to understand how local-scale processes vary across scale (e.g., how reservoir population dynamics are impacted by human land-use, or how poverty translates to high-risk human behavior etc.). The outcomes of this research are expected to provide critical insights into patterns and processes of LASV spillover, contribute to global understanding of the virus, and inform targeted intervention strategies to mitigate the impact of LASV in West Africa. Understanding these processes within human-driven environments is of paramount importance, given the rapid and unprecedented rate at which the natural world becomes human-dominated and the associated rise of emerging infectious diseases at the human-animal interface. The COVID-19 pandemic has been a stark reminder of our failure to understand these systems fully and make reasonable predictions of zoonotic spillover. Further work in this area will improve our ability to predict and ultimately minimize spillover risk in human populations globally.

### Strengths and limitations

This study integrates ecological, epidemiological, and anthropological data to examine Lassa virus dynamics within coupled human–rodent–landscape systems. Predictive modeling is used to synthesize heterogeneous data streams and to support hypothesis generation regarding transmission processes. The study is conducted in Nigeria, a region where LASV is endemic, and incorporates participatory research components to support ethical implementation and contextual interpretation.

Integrating data across disciplines presents analytical challenges related to differences in measurement scales, spatial and temporal resolution, and underlying assumptions. While the study generates fine-scale, place-based data, there are limitations in extrapolating findings to broader regional contexts, and additional modeling assumptions will be required to assess scalability. Study implementation may also be influenced by external factors such as logistical constraints, accessibility of remote areas, and changes in the public health or policy environment.

## Data Availability

No data are associated with this article.
