# Supplementary material for: Scaling Lassa Virus Dynamics within Anthropogenic Ecosystems (SCAPES) Study Protocol: a mixed-methods observational cohort study of humans, rodents, and landscapes in Nigeria
Source: Wellcome Open Res. 2026 Jul 28;11:125. Originally published 2026 Feb 16. [Version 2] doi: 10.12688/wellcomeopenres.25937.2 (PMC13554831; doi:10.12688/wellcomeopenres.25937.2)
Supplement: Peer review history

# Reviewer report: Reviewer response for version 2

**DOI:** 10.21956/wellcomeopenres.29257.r162422

**Published:** 2026-07-29

**Authors:** Joel Henrique Ellwanger

**Affiliations:** 1 Department of Genetics, Universidade Federal do Rio Grande do Sul - UFRGS (Ringgold ID: 124596), Porto Alegre, State of Rio Grande do Sul, Brazil

The authors have satisfactorily addressed all of my comments. I have no further requests.

Is the study design appropriate for the research question?

Yes

Is the rationale for, and objectives of, the study clearly described?

Yes

Are sufficient details of the methods provided to allow replication by others?

Partly

Are the datasets clearly presented in a useable and accessible format?

Not applicable

Reviewer Expertise:

Genetics; Environmental sciences; Infectious diseases

I confirm that I have read this submission and believe that I have an appropriate level of expertise to confirm that it is of an acceptable scientific standard.

# Reviewer report: Reviewer response for version 1

**DOI:** 10.21956/wellcomeopenres.28548.r148273

**Published:** 2026-04-23

**Authors:** Gebbiena M. Bron

**Affiliations:** 1 Norwegian Veterinary Institute, Ås, Norway

In short, this is a study protocol to better understand the drivers of Lassavirus (LASV) exposure in humans. The study combines longitudinal data of landscapes, rodents and humans in three focal sites in Nigeria to develop empirical data to inform integrated models to make predictive disease risk maps. The project plans to validate the predictions (e.g., rodent abundance, LASV prevalence) outside of the focal sites. The aim is to bridge the gap between local (fine-scale) observations and broad-scale (policy level scale) risk maps, giving risk maps a stronger underpinning. The model(s) will be used to co-design interventions aimed at reducing LASV burden. 

 The protocol uses mixed methods in an ambitious way, and the outcomes may provide more general insights into rodent-borne zoonotic pathogen spillover. 

 Due to the multidisciplinary nature of the protocol, I cannot assess all components at an equally thorough level as I would have liked. Throughout this review I will remark where I am ignorant of the details which may help the authors in further clarifying their approach to enhance reproducibility by other teams.  

**Is the rationale for, and objectives of, the study clearly described?**

 The rationale is clear and well explained.  

- LASV is a good study system as there is limited human-to-human transmission. There is a seasonal pattern, but also continuous transmission. However, many parts of disease transmission are not fully understood, which could be a challenge in collecting the ‘right’ data for the local scale model that can be translated onward. A few more details on the main reservoir, i.e. duration of infection/infectivity and lifespan, would be helpful for becoming familiar with the system quickly. 
- LASV exposure in Nigeria is quite variable, allowing for site comparisons within country. 

- The mismatch in local observations and global / national interventions is well received by me as a reader, but examples from LASV or other systems depicting these specific mismatches more detailed may strengthen the argumentations. However, this is not essential.  

- The study would benefit from describing the potential levels of interventions and how this links to the model scales, from who is targeted with an intervention e.g., individual behavior of target groups, blanket intervention for whole populations, community services, landscape structure etcetera. Is the intervention preventative or re-active? Depending on the reader’s reference frame they may consider any type of management tool from road development, trash collection, rodent control, food / hand hygiene, or introduction of alternative protein sources as possible interventions (as prompted by paragraphs 3 of the introduction). Some clarification on the project’s ideas of possible interventions would be useful, also for the rational for sample size estimates. 

- In the section ‘aims and objectives’: The goal of the study leads to the aims, but the goal on its own does not explicitly link to aim 1 (identifying opportunities for disease control) and aim 3. I would suggest adding to the goal statement that the forecasting tool will be used for individual and community(?) level intervention design.  

**Is the study design appropriate for the research question? **

- The study design is moderately clear; there are three focal sites / study areas each in a different state. For each focal site / study area, there are at least three villages included. These nine villages are included in the baseline assessment: household level questionnaire and serosurvey of man, women and one or more children in the household. But one focal village is used for drone-based remote sensing, geo-ethnography and possibly rodent trapping. The duration of the study can be inferred, but a timeline would simplify understanding what happens when and where.  

- If the study design is appropriate for the research question, is hard to assess as the sample size calculation is minimal and does not seem to address the study components and modelling approaches. That said, this is a complex design whereby sample size calculations are challenging. However, I would appreciate separate section(s) addressing sample size calculations linked to the planned analyses.  

My main concerns, for which I would like to see sample size explanations: Between and within site variation - I suspect that the within focal site variation needs to be substantial to allow for between focal site extrapolation at a larger scale, which I think will be desired to be able to distinguish between the larger underlying drivers for which the sites vary. But here I am not well versed enough in the multi layered modelling approach proposed. In addition, I am uncertain if GPS tracking and interviewing 90 people (30 of each group – male, female, kid – 10 of each per focal village) is sufficient to capture the desired ‘motifs’, especially in the context of the proposed dimensionality reduction. However, the latter is far from my expertise, and I am looking forward to seeing what happens in this process, if we do not accidentally end up with the ‘known’ high level risk map parameters.  

 Furthermore, the design of the rodent study misses some details (see below) and I am uncertain how trap results are linked to density. Based on the research questions, a sense of population dynamics is desired (e.g., relative abundances over time) as well as spatial (and temporal) activity overlap between rodents and people. Here I do not know enough about the rodent lifespans and reproduction patterns to be able to assess if the quarterly trapping for 4 nights with 450 traps is sufficient. In regards to the underlying research question, and aim to map overlap in human and rodent activity, I wonder if a camera trap approach would have been a useful addition to assess rodent activity (and density) as trap results will be heavily influenced by local food availability and not necessarily reflect where rodents spent most of their time or are most dense. The trap component is essential to estimate LASV prevalence in animals, but it would be helpful to provide sample size estimates for this component as well. 

  

- Underlying this complex and thorough design lingers the gap in understanding of viral dynamics. This seems to be a liability for interpreting the data. This gap should be discussed in the final paragraph of the manuscript. For example, based on the introduction there is a possibility for substantial transmission due to environmental contamination (e.g., surfaces and food) and, I suspect, viral particle survival will be greatly impacted by surface type, temperature, relative humidity and UV (i.e. local weather conditions) and it is unclear to me if/how that will be incorporated.  

**Are sufficient details of the methods provided to allow replication by others.**

 To make this study protocol detailed enough for replication, many supplemental pages will need to be added, which seems excessive. In general, this study protocol describes a clear framework for others to implement. The model details are very detailed in some places and more generic in other places, for example there is variation in the detail of potential covariates included or specifically what built-up area subclasses will be considered. This could be further addressed, but is not essential for me at this point. 

 I would expect more clarity on the rodent trapping protocols: Will 100 (10x10 grid) or 50 traps be used in the forest – agricultural matrix? How are trap locations spaced (10 or 100 m apart)? Are both trap types placed at the same trap location or randomly?  How are 400 traps placed in the village? Are animals killed, because organs can be collected or is that an omission in the description as removal of animals is also not addressed in the model and potential impact on transmission dynamics. 

 In the drone flight description, the speed of the drone, the area covered per image, and total intended area to be captured may be helpful for quick –gut feeling- evaluation how long this may take. In theory, this can be calculated based on 25 m flight overlap leading to 70% image overlap combined with the pixel dimension and taking a picture every 2 seconds, but I was uncertain if my high school algebra was actually accurate. I was also uncertain where the buffer will be placed around (the landscape types?).  

 In the Aim 2 section on validation surveys and model calibration, the intended patch size for hotspots is unclear to me. Will upscaling happen at state level or at random octagons of different sizes? Here I also wonder, why rodent composition should be assessed (this seems rather ambitious and I would expect a quite large acceptable error margin). In addition, I wonder if a more targeted effort capturing the main reservoir hosts for abundance and LASV prevalence would suffice (if possible at all). Such an approach, if possible, could further reduce the needed number of trapnights. Details about the survey in this section are more detailed than in the first mention of the survey and I would suggest moving them to the first survey round description. 

**Are the datasets clearly presented in a useable and accessible format?**

 No data is presented in the protocol. I would appreciate some details of how FAIR data / wellcome trust protocols are proposed to be implemented, e.g., where and how data and code will be shared.  

**Overall remarks and considerations **

1. More specific descriptions of the risks of interest would be helpful. Risk is frequently used, but it is sometimes unclear what concept it refers to:  

- seroconversion as a proxy of the probability of getting exposed to Lassa virus, and  

- disease cases as a proxy for the probability of getting Lassa fever (i.e. getting exposed, getting sick and being registered as sick).  

It would be helpful for the reader to understand what risk is referred to specifically when the term is  used.  

 In addition, the study design captures the core components of risk, but I think it helps to be explicit  that risk (e.g., seroconversion to LASV) depends on the environmental hazard (the ‘density’ of viral  particles in the environment including virus in rodents) and probability of exposure to this hazard  (human behavior / time spent in exposed areas). 

1. This is a study protocol; thus, future tense is expected. However, the project is already on the way and past tense has slipped into the methodology. I would be fine with the tense being consistent throughout the protocol or the tense matching the current state of the study protocol, finished tasks (e.g. baseline assessment) in past tense, ongoing work in present tense, and planned work in future tense. 
2. Throughout the protocol zoonotic, transmission, burden / challenge are used, here a basic disease ecology mindset with human disease as the outcome is expected from the reader. Especially in the abstract adding clarifying wording will make the protocol more accessible for the less disease ecology focused reader. For example: ... critical challenge to human health; Pathogen exposure / disease burden? risk maps are ...; local-drivers of pathogen transmission (and later in the text LASV spillover or LASV transmission). 
3. In the final section of the study protocol, the link with COVID is made, this was a distraction to me and didn’t seem like the main challenge that the work is taken on right now. This study aims to better understand rodent-borne zoonotic spillover, but the major gain, I perceive, will be bridging between the local and national (or even higher) level intervention design. This may reduce the probability for spillover events becoming pandemics, yes, but the main gain – I think – is reduction in the burden of rodent-borne zoonotic diseases. 
4. For aim 3, I wonder if intervention co-design should be approached more broadly. This is briefly discussed in the methodology, where win-win interventions will likely be suggested. I wonder if the team also wants to mention the unintended potential consequences, and maybe even elucidate how interventions could, or will, be followed up (design of actionable interventions that can be evaluated).
5. Figure 2 is not legible in the printed version, and the arrows are not recognizable. I wonder if a simplified figure would be more informative, and the full figure should be left online only.  
6. For figure 4, I wondered about population density when I saw the occurrence map. Would average cases / 100.000 people / year be a feasible map to make? 
7. Based on the subtle wording on ‘mechanistic underpinning’ and ‘hypothesis generation regarding transmission processes’, I wonder if a more SIR type model is envisioned in this project of rodents and surfaces/environments. However, I did not see the infectious state of the environment explicitly mentioned in the model descriptions. If it is captured in one of the general model terms, I would suggest providing a brief explanation as this study does seem to have the data to build a detailed spatial – temporal transmission model. 

Looking forward to reading the updated version including the timeline, sample size calculations, and additional details on rodent trapping and modelling.

Is the study design appropriate for the research question?

Partly

Is the rationale for, and objectives of, the study clearly described?

Yes

Are sufficient details of the methods provided to allow replication by others?

Partly

Are the datasets clearly presented in a useable and accessible format?

Not applicable

Reviewer Expertise:

Rodent-borne and vector-borne (zoonotic) diseases.

I confirm that I have read this submission and believe that I have an appropriate level of expertise to confirm that it is of an acceptable scientific standard, however I have significant reservations, as outlined above.

# Response

**Published:** 2026-07-16

**Authors:** Sagan Friant

**Affiliations:** Anthropology, The Pennsylvania State University, University Park, Pennsylvania, USA

We are grateful to the reviewer for an exceptionally thorough and constructive report. The comments on sample size, rodent trapping detail, environmental transmission, and the conceptual definition of risk have materially improved the protocol, and we appreciate the reviewer's candour in identifying where their own expertise was a limiting factor — this made it easier for us to see where our description was doing too much work implicitly. We have grouped our responses thematically, following the structure of the review.

**Introduction and rationale** We have added biological context on the reservoir. Longitudinal demography of *M. natalensis* derives largely from agro-ecological studies in East Africa and Guinea, and we now state an approximate mean wild lifespan of 3 months. On infection dynamics, horizontal transmission typically produces acute infection followed by a period of viral shedding, whereas vertical transmission is hypothesised to result in chronic, lifelong infection. We have been explicit that variation in infectiousness across a host's lifespan remains a key unknown in Lassa ecology and have included laboratory-observed PCR detection windows to give readers a baseline for the system's temporal dynamics. On the mismatch between local observation and national or global intervention, the reviewer has identified the masking effect of administrative aggregation, and we have made this concrete in the Introduction: national surveillance metrics routinely obscure acute spatial and temporal heterogeneity at State and LGA levels, such that a low-risk national forecast may entirely miss a localised surge in a single LGA. This has direct implications for when and where interventions are targeted (such as risk communication or vaccines, if and when they become available), and is the mismatch this project seeks to resolve.  We agree that the protocol was insufficiently explicit about what "intervention" means here, and that this ambiguity also undercut our sample size rationale. Our study is designed to identify modifiable pathways within the Lassa DAG, and we now describe interventions at three scales in the Introduction: individual and household (rodent-proof food storage, improved housing construction, household rodent control); community (targeted rodent control campaigns, health literacy); and regional or national (evidence-based behavioural and educational programmes tailored to high-risk socio-ecological motifs). These are preventative rather than reactive, which we now state directly. Relatedly, we have revised the goal statement so that it explicitly links the forecasting tool to intervention design, establishing a structural bridge into Aim 3 that the original phrasing left implicit.

**Study design, timeline, and sample size** We accept that the original protocol did not make the sequence of activities legible. We have added a study timeline setting out what happens when, where, and by whom across the baseline, longitudinal, and validation phases, and we have consolidated sample size justification into a dedicated section rather than distributing it across the aims. For the baseline serosurvey, we now present formal power calculations. Using established Lassa seroprevalence tiers (high >40%, medium 10–20%, low <5%), a sample of 180 individuals per category provides 80% power (α = 0.05) to detect differences between risk levels. We also describe the systematic sampling approach (approaching every *n*th household) and the intra-household quota system (adult male, adult female, adolescent, child) designed to prevent demographic sampling bias. For the GPS cohort, we take the reviewer's scepticism seriously and want to be straightforward about the logic. Classical frequentist power calculations sit awkwardly with the spatial autocorrelation inherent in tracking data, and we do not claim otherwise. The cohort (30 households per village, ≈90 individuals) is instead parameterised to achieve thematic and occupational saturation. In rural subsistence settings, spatial behaviour is constrained by a highly conserved set of livelihoods — cultivation, transit, peridomestic processing — and tracking 30 households captures the dominant motifs of community space use while sitting at the logistical ceiling for intensive daily device recovery and community sensitisation. We have stated this reasoning, and its limits, explicitly rather than implying a power basis that does not exist. The reviewer's concern about within- versus between-site variation is well placed, and our answer rests on site selection rather than sample size alone. Candidate villages were identified from OpenStreetMap using a bespoke site-selection tool and stratified across a gradient of postulated spillover drivers, with land cover (ESA WorldCover) characterised within a 2 km radius. We targeted medium-sized villages (100–500 households) and excluded sites with >60% forest cover or >20% built-up area. Final selection was checked to ensure no systematic bias relative to other regional settlements, and to confirm that sampled villages spanned local variation in cropland and grassland proportions. The design therefore samples a genuine spectrum of spillover risk across high-, medium-, and low-risk tiers rather than opportunistically targeting known hotspots — which is what makes between-site extrapolation defensible. We have clarified this process in the Study site and population and Interface modelling and scaling sections. Finally, the reviewer raises the possibility that dimensionality reduction may simply recover the "known" high-level risk map parameters. This is a real risk that we have designed against. We apply Bayesian regularisation via shrinkage priors, so that a fine-scale, drone-derived motif is retained only if it contributes statistically independent predictive skill over the broad-scale baseline. This is now described in Data integration and dimensionality reduction.

**Rodent ecology and trapping** We have expanded the rodent methods considerably and specified the trapping protocol in full. In the agricultural/forest matrix, each village receives six 10 × 10 grids deployed at half density (50 traps per grid at alternating stations, ~13 m spacing) to maximise spatial coverage while limiting inter-trap interference. In the peridomestic environment, approximately 210 traps per village are deployed: six traps inside each of the ~30 enrolled geo-ethnography households (180) plus 30 outdoor traps across shared village compounds. Both Sherman and Tomahawk live traps are used to capture the range of small-mammal body sizes. Trapping runs for four consecutive nights per session, quarterly, giving 2,040 trap-nights per village per session and a cumulative effort of 48,960 trap-nights over the 24-month study. Captured animals are euthanised — the reviewer correctly inferred that this was an omission rather than an ambiguity — as lethal sampling is required to collect spleen and liver for high-sensitivity RT-PCR. Trap density and peridomestic allocation were calibrated during piloting to match the processing capacity of our mobile field laboratories. On the relationship between trap results and density, we agree that absolute density (individuals per hectare) is not estimable without closed-population capture–mark–recapture. We therefore use hierarchical N-mixture models (spAbundance) to estimate latent relative abundance, modelling species- and site-specific detection probabilities explicitly from the regularised grid design. We now describe this as relative abundance throughout, rather than implying density. On temporal dynamics, quarterly deployments are designed to capture seasonal reproductive pulses rather than cohort survival, and we now say so. We supplement this with age structure across cross-sectional sampling events, allowing identification of juvenile recruitment surges following harvest. We also note that, given the *r*-selected reproductive capacity of *M. natalensis* and rapid compensatory immigration from the surrounding agricultural matrix, temporary lethal off-take is not expected to meaningfully perturb local population equilibria; we have added the relevant citation and flagged this assumption explicitly rather than leaving removal effects unaddressed in the model. The camera trap suggestion is a good one and we have adopted it in modified form: exploratory camera deployments at a subset of trapping stations will allow us to compare the number of rodents interacting with traps against those actually captured, giving a handle on seasonal trap-shyness and on the food-availability bias the reviewer identifies. We are grateful for the suggestion. On whether full community composition is necessary: we think it is, and the reason is inferential rather than descriptive. Synanthropic competitors — particularly *Rattus rattus* and *Mus musculus* — exert strong structural influence on *Mastomys* peridomestic occupancy. An absence of *Mastomys* inside a household is uninformative unless we can establish whether the domestic niche is genuinely vacant or is competitively saturated by non-competent hosts. The detection of LASV by PCR in sympatric rodent and shrew species reinforces the case for broader sampling. We have added this justification to Rodent sampling and diagnostics.

**Viral dynamics and the infectious environment** The interface model as specified is a hierarchical spatial model of exposure risk, not a mechanistic compartmental (SIR-type) transmission model, and it does not contain an explicit infectious-environment compartment. We considered parameterising one. Indirect transmission via contaminated grain stores, mounded crops, peridomestic surfaces, and aerosolised dust is a documented exposure pathway, but the environmental survival limits of LASV virions across heterogeneous rural surfaces — as a function of surface type, temperature, humidity, and UV — remain poorly characterised, and we do not measure environmental persistence directly. An explicit decay compartment would therefore rest on substantial parameter speculation, and we judged that a poor trade against the appearance of mechanistic rigour it would confer. Our approach instead treats environmental contamination as an unobserved latent effect: spatial random fields within the hierarchical models absorb unmeasured, site-specific environmental hazard variance without over-parameterising biology we have not measured. We have added a paragraph to the final section of the Discussion stating plainly that the absence of environmental viral dynamics is a limitation on interpretation, identifying it as a priority for future work, and noting that the longitudinal rodent infection data generated here would support a more explicitly mechanistic transmission model should the environmental persistence parameters become available.

**Aim 2: scaling, validation, and section organisation** We recognise the ambiguity around upscaling and have revised Interface modelling and scaling to provide more details. Prediction will not be projected onto administrative boundaries or irregular patches, but onto a regular 1 km² raster grid. This aligns with standard macro-ecological covariate layers (ESA WorldCover, CHELSA) and avoids the spatial distortion associated with the Modifiable Areal Unit Problem, while remaining fine enough to support operational risk stratification. On the reviewer's suggestion to relocate survey detail: We take this to refer to the participatory methods, which were described in greater procedural detail under the Aim 2 validation surveys than at their first mention. We agree this meant the reader encountered the framework before having the context to interpret it. We have therefore moved the descriptive detail up to the participatory rural appraisal section where these methods are first introduced, so that the approach is intelligible at first pass without requiring the reader to look ahead — or to consult the associated methods paper. The validation section now describes only what is distinctive about the streamlined "light-touch" adaptation.

**Data governance and FAIR implementation** Analytical pipelines, spatial covariates, and scripts will be version-controlled under the RISK Lab GitHub organisation, with partner repositories forked centrally at the point of manuscript submission to ensure link stability; analytical milestones and published codebases will be archived to generate version-specific DOIs; and SOPs for field and laboratory work will be hosted in the repository's open documentation. Given the sensitivity of high-resolution GPS tracks, raw coordinates will not be published: movement data will be de-identified by affine transformation into a local coordinate frame (bounding box translated to origin, random rotation applied), preserving the relative spatial and topological structure needed to reproduce the analyses. Because this transformation is distance-preserving, we describe it as de-identification rather than anonymisation and withhold the transformation parameters. These details are now in the Data availability section.

**Terminology: risk, hazard, and exposure** The reviewer is right that our use of "risk" was doing too much work. We now define the primary endpoint as the probability of pathogen *exposure*, indexed serologically by anti-LASV IgG, and distinguish it explicitly from the probability of clinical *disease*. Our own baseline data make the reviewer's point for us: among enumerated individuals, none reported a historical clinical diagnosis of Lassa fever. We have added a sentence to Interface modelling and scaling clarifying that infection is not a case. We have also adopted the reviewer's framing directly, and it improved the manuscript. Risk is now explicitly conceptualised as the joint product of environmental Hazard (the spatiotemporal abundance of infected rodents and environmental viral load) and Exposure (human behaviour, movement, and time spent in contact with that hazard). This framing is stated in Interface modelling and scaling and now carries through the Discussion.

**Tense, spelling, and accessibility** We have harmonised tense to match the current state of the study, as the reviewer suggests: completed work (the baseline assessment) in past tense, ongoing work in present, and planned work in future. We have also standardised spelling conventions throughout. Following the reviewer's specific suggestions, we have revised the abstract to be intelligible to readers outside disease ecology — referring to a critical challenge to human health, to pathogen exposure and disease burden, and to local drivers of pathogen transmission, in place of terminology that presumed a disease-ecology frame.

**Discussion and figures** The principal gain of this work is the reduction of endemic rodent-borne disease burden and the bridging of local and national intervention design; pandemic preparedness is a secondary implication and framing it around COVID-19 in particular was a distraction that we have removed. On the unintended consequences of interventions, the reviewer raises something we regard as a genuine research imperative: uncoordinated rodent culling can trigger compensatory breeding and accelerated juvenile immigration, with the potential to increase rather than reduce risk. Formally parameterising these feedback loops sits beyond this data-collection protocol, but investigating such systemic backfires through scenario-based simulation is a core objective of the Aim 3 co-design framework, and we have said so explicitly, together with a note on how co-designed interventions could be structured so as to be evaluable. Figure 2 has been redesigned as the fully articulated 60-node DAG was indeed illegible in print. We have included a simplified modular causal diagram organised across three tiers — upstream drivers, the socio-ecological interface, and clinical trajectories — which preserves directional structure and the overarching One Health logic for clarity for print readers, while the complete network is retained as a figure panel for online readers and on the project website, where readers can zoom and interrogate individual edges. On Figure 4, we should clarify what the panel shows. Panel A depicts the predicted *probability of occurrence* of Lassa fever, derived from broad-scale models of reported clinical cases, rather than a continuous incidence rate. Translating these 0–1 probabilities into average cases per 100,000 per year is not possible without additional modelling. A separate descriptive map of raw reported LGA case rates would be feasible, but would principally capture passive surveillance ascertainment rather than the underlying socio-ecological transmission hazard our sites were selected to investigate — which is the very conflation the reviewer elsewhere asks us to avoid. We have therefore clarified the figure legend and the accompanying text rather than adding the layer.

# Reviewer report: Reviewer response for version 1

**DOI:** 10.21956/wellcomeopenres.28548.r148274

**Published:** 2026-03-05

**Authors:** Joel Henrique Ellwanger

**Affiliations:** 1 Department of Genetics, Universidade Federal do Rio Grande do Sul - UFRGS (Ringgold ID: 124596), Porto Alegre, State of Rio Grande do Sul, Brazil

The study protocol by Friant *et al*. employs Lassa fever, a rodent-borne disease, as a model for understanding the local factors that determine the risk of zoonotic spillover events in Nigeria. Utilizing a One Health approach, the distinguishing feature of this study is its focus on situating the spillover problem at the local level within the broader context of high-risk areas.

 Given the persistent challenges posed by zoonotic diseases and the risk of spillover events in a world increasingly impacted by human activity and its consequences (e.g. land-use changes, pollution, climate change), this protocol describes a potential relevant approach to the prevention of new disease cases and contributes to the investigation of optimal strategies for outbreak prevention. However, certain points within the manuscript could be refined, as I detailed below:

 - Introduction (page 4): The authors state: “ *Zoonotic spillover – the process by which a pathogen is transmitted from an animal to a human host – is a poorly understood phenomenon*”. I suggest modifying this sentence to emphasize that the term “zoonotic spillover” typically refers specifically to the initial process by which a zoonotic pathogen is introduced into a new vertebrate host/population, such as humans. As currently written, the phrase describes a classic concept of “zoonosis” or “zoonotic infection”, which are distinct from “zoonotic spillover”. I recommend that the authors review their usage of these terms throughout the manuscript to ensure semantic coherence in their application.

 - Methods: Rodent reproduction is subject to fluctuations driven by food availability, which is, in turn, linked to temperature variations, extreme weather events, and seasonality. Such fluctuations in rodent populations influence infection risk. It is not clear how differences in reproductive rates will be accounted for in the study. I suggest that the authors clarify this point (I believe this factor may have been considered and is indirectly addressed in the work, but this is not evident in the manuscript).

 - Methods, “ *household-level factors (e.g., housing materials, wealth)*” (page 11): The presence of refuse (trash/litter) is one of the most critical factors for the aggregation of rodents near homes, as it provides a food source (organic waste) and shelter (inorganic waste) for these animals. How will this specific factor be considered in the study?

 - Methods: In addition to direct encounters with rodents, how will the presence of rodent feces in domestic and peridomestic environments be considered in the study? Contaminated feces represent a significant risk factor for infection via aerosols.

 - Discussion: The authors should add references (citations) to the text in this section wherever claims, specific information, or examples are presented. For instance, the phrase “ *localized studies revealing fine-scale disease processes*…”; Which specific studies are the authors referring to? The entire section is written without references, yet the information cited is derived from knowledge built upon previous studies and filed projects.

 - Discussion: The discussion could also specifically address how this study will contribute to reducing Lassa fever cases in the study region.

 Overall, the protocol is well-structured and appears robust. The manuscript has scientific merit and deserves to be indexed in its final form.

Is the study design appropriate for the research question?

Yes

Is the rationale for, and objectives of, the study clearly described?

Yes

Are sufficient details of the methods provided to allow replication by others?

Partly

Are the datasets clearly presented in a useable and accessible format?

Not applicable

Reviewer Expertise:

Genetics; Environmental sciences; Infectious diseases

I confirm that I have read this submission and believe that I have an appropriate level of expertise to confirm that it is of an acceptable scientific standard, however I have significant reservations, as outlined above.

# Response

**Published:** 2026-07-16

**Authors:** Sagan Friant

**Affiliations:** Anthropology, The Pennsylvania State University, University Park, Pennsylvania, USA

We thank the reviewer for their positive assessment and for their constructive points that have sharpened both the framing and the methodological detail of the protocol. We address each below.

**1. Definition of zoonotic spillover.** We agree that this term has been variably used and defined in the field. We now ground our definition in Plowright et al. (2017), where spillover is defined mechanistically as transmission of a pathogen from a reservoir population to a novel host, requiring the alignment of multiple ecological and epidemiological barriers. Because those barriers can align repeatedly, spillover is a recurrent phenomenon — particularly for endemic pathogens such as Lassa virus, Nipah virus, and the hantaviruses. Restricting the term to a singular historical event risks conflating spillover (cross-species transmission) with host shift or disease emergence (subsequent evolutionary adaptation and sustained onward transmission). Lassa virus spills over from rodents to humans many thousands of times each year, and it is precisely the socio-ecological drivers of these recurrent events that our study examines.

**2. Rodent reproductive fluctuations.** The reviewer is right that demographic pulses strongly influence infection risk, and we agree this needed to be made explicit. Our design does not use capture–mark–recapture, so we do not estimate absolute population size. We do, however, collect extensive demographic data from captured individuals — age, development, fecundity, and parity (including evidence of fetuses and recent pregnancy) — and our Bayesian joint species distribution models estimate latent abundance, allowing population fluctuations to be accommodated rather than assumed away. We have added fecundity and parity to the list of demographic variables in Rodent sampling and diagnostics and have added text to the modelling section stating that these metrics will be used to account for seasonal reproductive pulses and their influence on transmission risk.

**3. Household refuse.** We accept this as a genuine gap in our systematic quantification. During participatory rural appraisals, communities consistently described organic waste around households as ubiquitous and highly variable, which makes standardized measurement across diverse waste-management practices difficult. Our design therefore prioritizes systematic quantification of indoor food storage practices — identified through community knowledge as the primary *controllable* exposure pathway — while capturing refuse contexts through complementary means: photographic documentation of household environments, structured surveyor observations, and drone-based mapping of prominent waste accumulation sites at village scale. This characterizes refuse qualitatively and spatially without claiming a precision we could not deliver for a variable community themselves described as seasonally dynamic. We have clarified this in Questionnaire and serosurvey and noted in Drone-based remote sensing that prominent waste sites will be identified and mapped. Recognizing that this is a key practice that may influence variation between villages, we have also explicitly described it as part of our light touch protocol.

**4. Rodent faeces and aerosol exposure.** We agree that contaminated faeces represent an important transmission hazard, and it is already integrated across several data streams — though this was not stated clearly enough in the original text. The individual questionnaire and the geo-ethnography surveys both document the presence, sighting, and handling of rodent faeces within the home and in food storage areas. We have added explicit text to this effect in Questionnaire and serosurvey.

**5. Citations in the Discussion.** A fair criticism. We have systematically reviewed the Discussion and inserted supporting citations throughout, including for broad-scale risk maps and associations (Redding et al.), the localised studies revealing fine-scale disease processes referred to in the passage the reviewer highlights (Mariën et al., 2018, 2020), the annual recurrence of Lassa fever outbreaks and the absence of a licensed vaccine, and the historical sampling bias that has concentrated surveillance in recognised hotspots.

**6. Contribution to reducing Lassa fever cases.** This is the central purpose of Aim 3, and the reviewer is right that the Discussion did not make the connection explicit enough. We have added a concluding paragraph setting out the operational logic: by identifying specific, *modifiable* socio-ecological risk factors across both hazard and exposure, the project returns empirical findings to communities and local stakeholders (including NCDC) in order to co-design targeted, culturally acceptable, and feasible interventions aimed at reducing human–rodent contact and, in turn, incidence.

# Reviewer report: Reviewer response for version 1

**DOI:** 10.21956/wellcomeopenres.28548.r148268

**Published:** 2026-02-28

**Authors:** Cyril Erameh

**Affiliations:** 1 Irrua Specialist Teaching Hospital, Irrua, Edo, Nigeria

**Manuscript:** *Scaling Lassa Virus Dynamics within Anthropogenic Ecosystems (SCAPES) Study Protocol*

**Article type:** Study Protocol

 Summary

 This protocol describes an ambitious, interdisciplinary, longitudinal study integrating ecological, epidemiological, anthropological, and spatial data to understand spillover dynamics of Lassa fever in Nigeria. The study combines participatory rural appraisal, drone-based remote sensing, geo-ethnography with GPS tracking, rodent ecology, serology, and Bayesian cross-scale modeling to bridge local transmission processes with broad-scale risk prediction.

 The protocol is conceptually strong, methodologically rigorous, and well aligned with One Health principles. It addresses an important gap in spillover science: linking mechanistic local processes to regional risk mapping.

 Major strengths

- Clear cross-scale conceptual framework grounded in causal inference (use of DAGs and Bayesian modeling).
- Innovative integration of fine-scale land cover mapping, human movement data, and rodent infection dynamics.
- Explicit out-of-sample validation design to address historical surveillance bias.
- Strong participatory and ethical framework, including community engagement and intervention co-design.

Points for clarification

1. **Longitudinal duration and timing:** Please clarify total follow-up duration and timing of serological sampling for incident infection.
2. **Serological assay performance:** Provide sensitivity/specificity estimates for the ELISA and describe how misclassification will be handled analytically.
3. **Power for longitudinal incidence:** Brief justification of expected seroconversion rates and event counts would strengthen the protocol.
4. **Model complexity and parsimony:** Outline safeguards against overfitting given high-dimensional data and modest cohort sizes.
5. **Data sharing and governance:** Clarify plans for data/code availability and handling of sensitive spatial data.

Overall assessment

 This is a high-quality, well-designed protocol that addresses a major question in zoonotic disease ecology using an innovative, cross-scale approach. The requested clarifications would enhance transparency but do not affect the scientific integrity of the study.

 Recommendation: Approve with minor revisions.

Is the study design appropriate for the research question?

Partly

Is the rationale for, and objectives of, the study clearly described?

Yes

Are sufficient details of the methods provided to allow replication by others?

Partly

Are the datasets clearly presented in a useable and accessible format?

Yes

Reviewer Expertise:

Lassa Fever and emergent pathogens, internal Medicine, clinical trials, and Neurology

I confirm that I have read this submission and believe that I have an appropriate level of expertise to confirm that it is of an acceptable scientific standard, however I have significant reservations, as outlined above.

# Response

**Published:** 2026-07-16

**Authors:** Sagan Friant

**Affiliations:** Anthropology, The Pennsylvania State University, University Park, Pennsylvania, USA

We thank the reviewer for their careful reading and for the generous assessment of the protocol. We are grateful for the clarifications requested, each of which has improved the transparency of the manuscript. We respond to each in turn.

**1. Longitudinal duration and timing.** The follow-up period corresponds to the funded SCAPES field period. Longitudinal geo-ethnography and rodent sampling will run over 24 months and replicate serological sampling of the human cohort is a single follow-up event at the conclusion of this observational period, capturing cumulative incident infections. We have stated the 24-month duration explicitly and clarified this temporal structure in the Geo-ethnography section, where it was previously left implicit.

**2. Serological assay performance.** We have added the sensitivity, specificity, and accuracy of the Panadea® LASV IgG assay to the Questionnaire and serosurvey section, together with a note on the distinction between dried blood spots (DBS) and whole blood. We anticipate some reduction in sensitivity arising from the DBS elution process, and we now acknowledge this explicitly as a caveat on prevalence estimates. On the analytical handling of misclassification, we have made a deliberate choice not to incorporate assay parameters as informative priors in the Bayesian models. Doing so would add computational complexity and widen uncertainty intervals around prevalence without materially altering inference on the spatial and socio-ecological risk factors that are the primary targets of the study. We felt it was preferable to state this reasoning openly rather than leave the decision unexplained.

**3. Power for longitudinal incidence.** This is a helpful prompt to be clearer about scope. The repeat serology within the geo-ethnography cohort (~90 individuals) is not designed or powered to estimate population-level incidence at the village scale. It is an exploratory, within-person component intended to link individual movement footprints and documented behaviours directly to infection outcomes. Because the aim is to characterise socio-ecological exposure profiles rather than to generate generalisable incidence rates, a formal power calculation for population-level seroconversion would not be the appropriate justification. We have added text to the Geo-ethnography section making this scope explicit and stating that the component is not intended to yield incidence estimates generalisable to populations lacking high-resolution behavioural data.

**4. Model complexity and parsimony.** We share the reviewer's concern. Alongside non-negative matrix factorisation for dimensionality reduction, our framework relies on regularisation within the Bayesian models — regularising priors (e.g. horseshoe or ridge penalties on regression coefficients) that shrink weakly informative predictors toward zero — combined with out-of-sample cross-validation to assess generalisability. Both safeguards are now stated explicitly in Interface modelling and scaling.

**5. Data sharing and governance.** Tabular baseline data will be openly available, and we now direct readers to the repository associated with our baseline serosurvey manuscript. Raw geographic coordinates from the geo-ethnography cohort will not be published. Spatial data will instead be de-identified by projecting each village's movement data into a local coordinate frame via an affine transformation: the bounding box is translated to an origin of (0,0) and a random rotation applied, preserving the relative spatial and topological relationships required to reproduce movement and behavioural analyses. Because this transformation is distance-preserving, we describe it as de-identification rather than full anonymisation, and the transformation parameters will be withheld. Analysis code will be openly available via version control, with archived releases deposited to obtain persistent identifiers. These details have been added to the Data availability section.
